# Supplementary material for: Unlicensed GS-441524-Like Antiviral Therapy Can Be Effective for at-Home Treatment of Feline Infectious Peritonitis
Source: Animals (Basel). 2021 Jul 30;11(8):2257. doi: 10.3390/ani11082257 (PMC8388366; doi:10.3390/ani11082257)
Supplement: Supplementary file 1 [file animals-11-02257-s001.zip › animals-1213658-supplementary.pdf]

FIP GS-441524 Study (Retrospective)

* Required

General information

1. What country do you live in?

2. What state/province do you live in?

3. What is your cats name?

General information about your cat

4. How old was your cat when they were diagnosed with FIP? Please respond with years and months. *

5. How old is your cat at the time of taking this survey? Please respond with years and months. *

6. What was the sex/neuter status of your cat at the time of diagnosis with FIP? *

Check all that apply.

- [ ] Male (unneutered)
- [ ] Female (not spayed)
- [ ] Neutered Male
- [ ] Spayed Female

7. What breed is your cat? *

8. Did you receive your cat from a breeder? If so, did you take any steps to notify the breeder that your cat passed from FIP, how familiar was the breeder with FIP, and how did the breeder respond during your interaction? If this question does not apply, please write "Does not apply".

Diagnosis and clinical signs of Generic FIP symptoms

9. What signs of FIP did you, your vet, or any parties observe in your cat? Please check all that apply. Note that neurological, ocular, and wet FIP specific symptoms will be covered further down in the survey. *

Check all that apply.

- [ ] Lethargy/Listlessness
- [ ] Decreased appetite
- [ ] Refusing to eat (anorexia)
- [ ] Weight loss
- [ ] Fever
- [ ] Enlarged abdomen
- [ ] Difficulty breathing or cough
- [ ] Increase in water consumption
- [ ] Increase in urinations
- [ ] Vomiting
- [ ] Diarrhea
- [ ] Bloody stool
- [ ] Yellow tinge to the skin, gums, and eyes (jaundice)
- [ ] Difficultly walking
- [ ] Vocalization/meowing (as if in pain)
- [ ] Hiding or avoidance behavior
- [ ] Liver Involvement (inflamed liver, granulomas on liver, signs of liver disease, etc.)
- [ ] Gastrointestinal involvement (issues with loose stools or diarrhea, vomiting, granulomas on liver, etc)
- [ ] Kidney involvement (inflamed kidneys, granulomas on kidneys, signs of kidney disease, etc.)
- [ ] Fluid involvement
- [ ] Neurological involvement

Other: [ ] ______________________

Did your cat experience any suspected stressors prior to development of FIP?

Known stressors include things such as recent vaccinations, additions of cats to home, significant change of scenary (such as moving), recent illnesses distinct from FIP, and so on.

Please note that this list is non-inclusive, and stressors for FIP are not totally known yet, so if you noticed other stressors that may have contributed, please feel free to let us know those as well.

Did your cat experience any suspected stressors prior to development of FIP?

Known stressors include things such as recent vaccinations, additions of cats to home, significant change of scenary (such as moving), recent illnesses distinct from FIP, and so on.

Please note that this list is non-inclusive, and stressors for FIP are not totally known yet, so if you noticed other stressors that may have contributed, please feel free to let us know those as well.

10. If any suspected stressors were present, please provide details, as well as the time period in which the stressor occurred.

11. Do you remember any of the diagnostic tests and the results your vet did that lead to the presumptive diagnosis of FIP? For example, a blood test, an FIP PCR, etc. If none, please write "None".

12. Does you cats have any other concurrent disease(s)? Check all that apply . *

Check all that apply.

- [ ] FeLV (feline leukemia virus)
- [ ] FIV
- [ ] Was diagnosed with cancer during the course of treatment for FIP
- [ ] Had cancer prior to developing FIP (in remission)
- [ ] Had cancer prior to developing FIP (is not in remission)
- [ ] Heart disease
- [ ] Diabetes
- [ ] Liver disease
- [ ] Hepatic lipidosis (feline fatty liver syndrome)
- [ ] Chronic kidney disease
- [ ] Irritable Bowel Disease
- [ ] Parasitic infection
- [ ] Asthma
- [ ] Epilepsy
- [ ] Gingivitis
- [ ] Feline Herpes Virus
- [ ] No other diseases
- [ ] Had pneumonia while having an FIP diagnosis (before treatment, during treatment, or in remission)

Other: [ ] ______________________

13. Did your veterinarian know about GS or GC antiviral treatment options for FIP? *

Mark only one oval.

- ( ) Yes
- ( ) No
- ( ) Other: ______________________

14. Did you treat with FIP antiviral therapy (ie, GS or GC?) *

Mark only one oval.

- Yes *Skip to question 19*
- No *Skip to question 15*

Cats untreated

15. Do you know about GS treatment?

Mark only one oval.

- Yes
- No
- Other: ______________________

16. If you answered "Yes" to the previous question, why did you not start GS therapy with your cat?

Check all that apply.

- [ ] I was unaware of the treatment
- [ ] My vet said it was not effective
- [ ] My vet refused to help and said it was illegal
- [ ] I was apprehensive of ordering a black market drug and giving it to my cat
- [ ] The cost was prohibitive
- [ ] I was unable to obtain any GS prior to my cats passing
- [ ] I elected to humanely euthanize
- [ ] I answered "No" to the previous question

Other: [ ] ______________________

17. Did your vet recommend any of the following treatment options?

Check all that apply.

- [ ] Prednisolone/Prednisone/oral steroids
- [ ] Depomederol/injectable steroids
- [ ] Mirtazapine/Mirtaz
- [ ] Vitamin B12 injections
- [ ] Polyprenyl Immunostimulant (PI) or VetImmune
- [ ] Antibiotics
- [ ] Cerenia (for nausea)
- [ ] Subcutaneous fluids
- [ ] IV fluids
- [ ] T-cyte/Proboost/Thymic Protein A supplementation
- [ ] Blood Transfusions

Other: [ ] ____________________

18. Do you have any anything you would like to add regarding GS or therapy for FIP, or any additional thoughts you would like to share?

____
____
____
____
____

Cats treated

19. How did you learn about GS treatment for FIP?

Mark only one oval.

- Internet search
- Facebook page
- A friend
- Your veterinarian directly told you
- Your veterinarian indirectly told you
- Scientific journal
- Other: ______________________

20. Did your initial veterinarian help you monitor your cat through the therapy, or did you have to find a new veterinarian?

Mark only one oval.

- Yes, my vet that diagnosed my cat willingly monitored my cat throughout the process
- No, my original vet that diagnosed my cat was uncomfortable monitoring my cat through the treatment, and I found a new veterinarian to monitor through the process.
- My cat was diagnosed at the emergency veterinarian, and I had to find another vet to monitor my cat throughout the therapy.
- Other: ______________________

21. Did you have help from a veterinary professional during treatment with GS? Check all that apply.

Check all that apply.

- [ ] No, I ordered the medication and just treated my cat myself
- [ ] Yes, my vet was aware that I was providing GS therapy to my cat at home, and monitored the progress throughout.
- [ ] My vet did recheck examinations and laboratory work on my cat, but was not aware that I was providing GS therapy to my cat at home.
- [ ] My veterinarian was extremely helpful, and played a role in administering the GS therapy.

Other: [ ] ______________________

22. Please estimate your total cost of treatment with GS therapy for your cat, and length of treatment. Such as only 84 days, or the initial 84 days and then another 6 weeks, etc.... (Do not include additional cost of veterinary bills, just your total cost of medication thus far)

____
____
____
____
____

23. Please estimate your total cost of veterinary care, including diagnosis of FIP, monitoring through the course of treatment, and monitoring after remission. (Do not included the cost of the medication here)

____

24. Did you experience any difficulty originally ordering your GS medication?

Mark only one oval.

- No
- Yes
- Other: ____________

25. Did you experience any difficulty re-ordering the GS medication?

Mark only one oval.

- Yes
- No
- Other: ____________

26. How many days after being diagnosed with FIP did you begin the GS therapy? Please estimate the amount of time to days.

____

Did you need to extend the initial treatment period, for any reason other than a relapse?

This question seeks to gather which cats extended treatment for reasons not relapse-related. Reasons may include insufficient progress on bloodwork (ie, low A/G ratio, low lymphocyte count, etc.), insufficient progress on symptoms (ie, lingering neurological deficiencies), just to be extra cautious, and other factors.

This question does NOT include treatment extensions due to a relapse. A relapsed cat may be defined as:
1. A cat that completes treatment and is under observation, but begins to display symptoms again.
2. A cat that has symptoms under control (ie, is showing no or recovering symptoms) and symptoms dramatically worsen.
3. A cat that develops a new form of FIP during treatment (for example, a cat receiving treatment for dry or wet FIP, which develops neurological or ocular symptoms).
Relapse-related questions will be asked later on.

27. Please share.

Check all that apply.

- [ ] I extended the initial treatment because my cat's bloodwork was insufficient.
- [ ] I extended the initial treatment because my cat was still showing symptoms.
- [ ] I extended the initial treatment to be extra cautious.
- [ ] No, I did not need to extend treatment.

Other: [ ] ______________________

28. If so, how many weeks did you extend the initial treatment period?

29. What else regarding learning about and acquiring GS therapy do you think is important for us to know?

Treatment

30. What brand of GS treatment did you use the most during treatment?

Mark only one oval.

- Aura/Spark
- Brava/Rainman
- Slayer/Miner
- Hero - White cap
- Hero - Blue cap
- Shire
- Mark's
- Andy's
- Ruby
- Pine
- Mutian
- Capella
- Blossom
- SAK
- Oscar
- CureFIP
- Unknown
- My cat did not survive long enough to begin treatment
- Other: ____________________

31. What form of GS did you use throughout treatment?

Mark only one oval.

- Injectable
- Oral
- A combination of both oral and injectable
- Other: ____________________

32. If you answered a combination of both oral and injectable in the previous question, please explain why both forms were used?

33. Did you switch brands of GS at any point during therapy? And if yes, why?

34. What did you choose the form of the GS medication that you are giving/gave? (Form is oral versus injectable) Please check all that apply.

Check all that apply.

- [ ] Cost
- [ ] Easy to obtain
- [ ] Easy to administer
- [ ] Your veterinarian recommended to give it this way
- [ ] Your admin recommended to give it this way
- [ ] You are unable to give your cat an injection
- [ ] You are unable to orally give your cat a pill
- [ ] Your cat only tolerated this form

Other: [ ] ____________

35. Did you ever miss or purposely skip a dose?

Mark only one oval.

- Yes
- No

36. If you answered yes to the previous question, please explain.

37. How often do you/did you give your cat the GS treatment most frequently?

Mark only one oval.

- Once every other day
- Once a day
- Twice a day
- Three times a day
- Four times a day
- Other: ____________

38. Do you recall how long it took for you to first notice improvements in your cat's condition, if at all, during the first course of treatment for FIP? For example, if your cat saw symptom improvement around day 9, you should select "About 2 weeks". If your cat saw symptom improvement around day 2, you should select "Within the first 3 days".

Mark only one oval.

- Within the first 24 hours.
- Within the first 3 days.
- About 1 week.
- About 2 weeks.
- By about the 4th week (within the first month).
- By about the 6th week.
- By about the 8th week (within the first two months).
- My cat unfortunately never saw symptom resolution.
- Other: ______________________

39. What clinical symptoms of FIP did you notice went away first when starting therapy with GS? Check all that apply.

Check all that apply.

- [ ] Appetite increased
- [ ] Normal water consumption
- [ ] Was able to walk better
- [ ] Was able to run better
- [ ] Was able to jump up onto things
- [ ] Eyes looked better/resolved
- [ ] No more vomiting
- [ ] Formed stool
- [ ] No more coughing
- [ ] No more sneezing
- [ ] Energy level improved
- [ ] No longer twitched without stimulation
- [ ] Seizures decreased in frequency
- [ ] Seizures stopped
- [ ] Was able to breathe comfortably
- [ ] No blood in stool
- [ ] No longer has yellow tinge to skin, gums, eyes (jaundice)
- [ ] Appears to be able to see better
- [ ] No longer vocalizing
- [ ] No longer has a fever
- [ ] No longer hiding and is more social

Other: [ ] ______________________

40. How many total weeks of first-round treatment did it take for your cat to stabilize to the point where he/she looked basically back to normal? For example, if your cat's condition was back to normal by week 4 and remained stable for the last 8 weeks of treatment, please respond "4". If your cat's condition looked back to normal by week 6 of the first treatment period, but relapsed and later passed, please respond "6". If your cat never stabilized, respond "Never".

____

41. Is there any additional information you would like to share with us about your cat's symptom presentation timeline, or anything additional you would like to note in the above question?

What was the starting dosage that you gave to your cat when you began therapy?

This number will ideally be provided in mg/kg.

Generally for dry or wet forms of FIP, this number tends to be 2, 5 or 6 mg/kg. For Neurological or Ocular FIP, the number tends to be 8 or 10 mg/kg.

If you are unsure of the dosage, but know "HOW MUCH" of the treatment you gave your cat, you can also provide the following:
If giving injections ALL of the following 3 numbers:
1) The mL of the injections of treatment you were giving at the beginning
2) The concentration of the treatment you were using at the beginning (usually, 12.5 mg/mL, 15 mg/mL, 16.5 mg/mL, 17 mg/mL, or 20 mg/mL) OR the "unofficial name" of the treatment you were using at the start (ie, "white cap hero" or "Aura 17"),
3) The starting bodyweight of your cat when you began treatment

If giving pills, ALL of the following 2 numbers:
1) The number of pills you were giving, per DAY at the beginning (ie, if you were using pills every 12 hours, this would be the total number of pills across TWO pill administrations),
2) The most detailed description and full name of the pills (for example, "Aura 12 hour pills"),
3) The starting bodyweight of your cat when you began treatment

42. Please describe.

What was the most recent dosage that you gave to your cat when you began therapy?

This number will ideally be provided in mg/kg.

Generally for dry or wet forms of FIP, this number tends to be 2, 5 or 6 mg/kg. For Neurological or Ocular FIP, the number tends to be 8 or 10 mg/kg. For relapsed cats, this number may be higher.

If you are unsure of the dosage, but know "HOW MUCH" of the treatment you gave your cat, you can also provide any of the following:
If giving injections ALL of the following 3 numbers:
1) The mL of the injections of treatment you were giving at the end,
2) The concentration of the most recent treatment you were using (usually, 12.5 mg/mL, 15 mg/mL, 16.5 mg/mL, 17 mg/mL, or 20 mg/mL) OR the "unofficial name" of the treatment you were using at the start (ie, "white cap hero" or "Aura 17"),
3) The most recent bodyweight of your cat,

If giving pills, ALL of the following 2 numbers:
1) The number of pills you were giving, per DAY at the beginning (ie, if you were using pills every 12 hours, this would be the total number of pills across TWO pill administrations),
2) The most detailed description and full name of the pills (for example, "Aura 12 hour pills"),
3) The starting bodyweight of your cat when you began treatment

43. Please describe.

44. If these two numbers differ, please briefly explain why, as well as how many dosage changes were made.

45. Did your cat ever experience any wet (effusive) FIP symptoms? Ie, fluid/effusions in or around the abdominal cavity, lungs, or heart. *

Mark only one oval.

- Yes *Skip to question 46*
- No *Skip to question 53*

Wet FIP Questions

46. Did your cat's diagnosis when you started treatment include wet (effusive) FIP?

Mark only one oval.

- Yes
- No
- Other: ______________________

47. If your cat's initial diagnosis included wet (effusive) FIP, about when did your cat's effusions resolve after starting treatment?

Mark only one oval.

- Within the first week.
- Within the first two weeks.
- Within the first 3 days.
- Within the first 3 weeks.
- Within the first 4 weeks.
- Within the first 6 weeks.
- Within the first 8 weeks.
- My cat's effusions never resolved.
- My cat's diagnosis did not include wet (effusive) FIP at the start of treatment; we only noticed it later on.
- Other: ______________________

48. Did your cat ever experience any of the following? Select all that apply.

Check all that apply.

- [ ] I noticed effusions during or prior to a relapse.
- [ ] I notice effusions that started early in the treatment period, but my cat did not start with effusions.
- [ ] The fluid went away and came back later.
- [ ] My cat started with effusions, but they went away and never came back.
- Other: [ ] ______________________

49. Where did your cat experience fluid/effusions? Select all that apply.

Check all that apply.

- [ ] Abdominal cavity (ie, ascites, fluid in the abdomen, fluid in belly, etc)
- [ ] Pleural (ie, fluid in lungs)
- [ ] Pericardial (ie, fluid in or near the heart)
- Other: [ ] ______________________

50. How many times did you get fluid drained or biopsied during the course of treatment?

____

51. Can you describe the fluid? Select all that apply.

Check all that apply.

- [ ] Yellow-tinged
- [ ] Orange-tinged
- [ ] Red-tinged
- [ ] White-tinged
- [ ] The fluid had no color.
- [ ] Clear (in addition to any colors noted)
- [ ] Opaque or difficult to see through (in addition to any colors noted)
- [ ] Translucent (not quite totally opaque, but not totally clear)
- [ ] Thick
- [ ] Thin
- [ ] I can't recall.

Other: [ ] ____________________

52. Did your vet run any tests on the fluid? Select all that apply. If you have the test results and are comfortable with sharing, please send them to FIPwarriorsStudy@gmail.com along with bloodwork, as per the question on "report upload".

Check all that apply.

- [ ] My vet did not test the fluid.
- [ ] FIP or FeCV PCR
- [ ] FIP or FeCV IPS
- [ ] cytology (looking at the chemical contents in the fluid)
- [ ] Rivalta Test
- [ ] My vet tested the fluid, but I cannot recall what tests were done.
- [ ] I cannot recall whether tests were done on the fluid.

Other: [ ] ____________________

Neuro/Ocular FIP Symptoms

Neurological symptoms include, but are not limited to:
1. ataxia/gait related issues, such as uneven/unsteady walking/running/jumping
2. hind leg weakness (dragging of the back legs)
3. Tremors, shaking, or erratic twitching
4. Seizures
5. granulomas in the brain
6. balance issues/issues with falling/stumbling

Ocular symptoms include, but are not limited to:
1. Cloudy eyes
2. Uveitis (inflammation or redness of the white part of the eye)
3. Iritis (inflammation, redness, uneven color, or other color changes of the colored part of the eye)
4. Uneven pupils
5. Blindness, either partial or complete
6. imperfect pupil shape
7. granulomas in the eye

53. Did your cat ever show any neurological, or ocular, symptoms, as above? *

Mark only one oval.

- Yes *Skip to question 54*
- No *Skip to question 63*

Neuro/Ocular FIP Detailed Questions

54. When you began GS treatment, did your cat's diagnosis include neurological/ocular FIP?

Mark only one oval.

- Yes, I began treatment for neurological/ocular FIP
- No, I began treating my cat for a different form of FIP
- Other: ____________________

55. Which symptoms do you recall seeing in your neurological/ocular cat? Select all that apply. If you cannot remember which symptoms led to a diagnosis of neurological/ocular FIP, please select "I cannot recall".

Check all that apply.

- [ ] My kitty's walking/gait was unsteady/uneven or not present (my cat was unable or did not attempt to walk).
- [ ] My kitty's running was unsteady/uneven or not present (my cat was unable or did not attempt to run).
- [ ] My kitty's jumping was hesitant, or not present (my cat was unable or did not attempt to jump).
- [ ] My cat experienced balance issues (for example, kitty falls over frequently, or is unable to stand on his front feet).
- [ ] My kitty would drag his/her legs, or showed instability in the hind legs (hind leg weakness).
- [ ] My kitty experienced seizures.
- [ ] My kitty experienced tremors or shaking.
- [ ] My kitty experienced twitching (either in spells or constantly).
- [ ] My kitty's eyes appeared cloudy or hazy.
- [ ] My kitty showed signs of uveitis (inflammation or redness of the white part of the eye).
- [ ] My kitty showed signs of Iritis (inflammation, redness, uneven color, or other color changes of the colored part of the eye)
- [ ] My kitty had uneven pupil sizes.
- [ ] My kitty was either partially or completely blind.
- [ ] My kitty had erratically shaped pupils (for example, the pupil had uneven edges).
- [ ] My vet told me my cat had ocular granulomas.
- [ ] My cat's eyes would dart back and forth in an uncontrolled way (nystagmus).
- [ ] My cat would tilt his head awkwardly.
- [ ] I cannot recall.

Other: [ ] ______________________

How would you best describe your cat's mental state?

Note that this question is focusing less on how much your cat is able to interact with the world, and more on his/her ability to be AWARE of the world.

For example, a cat may have substantial neurological problems making him/her unable to actually interact at a normal level, but still display responsiveness to things such as being pet, the owner sitting next to him/her, or a laser/toy darting in front of his face (even if he/she is unable to play).

A cat with reduced mental state will be unaware of his/her surroundings (such as not moving his/her eyes or head to track objects in his/her field of view, looking "glassy-eyed", not responding to being pet, etc.). A cat in this state may be described as "catatonic" or "reduced response"/"non-responsive".

56. Please describe.

Mark only one oval.

- My cat was alert and responsive to my presence, or appeared to want to interact with the world, despite his neurological/ocular deficiencies.
- My cat was less alert and responsive than usual to my presence or other forms of stimulation (such as to toys or other feline family members), and seemed slightly less aware of what was going on around him than usual.
- My cat seemed practically unaware of my presence or other forms of stimulation (such as to toys or other feline family members), and seemed virtually unaware of what was going on around him/her.
- Other: ______________________

57. Which symptoms do you remember noticing improved first in your cat's neurological/ocular condition specifically? Please use your best judgment. If you cannot remember, please select "I cannot recall".

Check all that apply.

- [ ] My kitty's walking/gait.
- [ ] My kitty's running.
- [ ] My kitty's jumping.
- [ ] My cat's had better balance (for example, falls over less).
- [ ] My kitty dragging of his/her legs and hind leg weakness.
- [ ] Seizures.
- [ ] Tremors or shaking.
- [ ] Twitching.
- [ ] Eye cloudiness/haziness.
- [ ] Uveitis
- [ ] Iritis
- [ ] Pupil unevenness.
- [ ] Blindness.
- [ ] Erratic pupil size.
- [ ] My cat's eyes darted back and forth less (less nystasgmus).
- [ ] My cat's head tilt.
- [ ] I cannot recall.
- [ ] My cat never saw resolution of any of his/her neurologic symptoms.

Other: [ ] ____________________

Do you recall how long it took for you to first notice any improvement in your cat's NEUROLOGICAL or OCULAR condition specifically, if at all, during the first course of treatment for neurological/ocular FIP?

In this question we want to specifically know how long neurological or ocular symptoms took to show signs of improvement, even if the cat's other symptoms may have shown improvement sooner than the neurological/ocular symptoms.

For example, if your cat presented with a reduced appetite (not a neurological/ocular symptom) and hind leg weakness (a neurological symptom), and your cat's appetite returned around day 4, but you did not notice improvement in the hind leg weakness until day 8, you should respond with 8 days (within the first 2 weeks).

If your cat only developed neurological/ocular FIP during a relapse, we want to know how long after you noticed the neurological/ocular deficiencies that you started to see improvement after treating the relapse.

58. Please describe.

Mark only one oval.

- Within the first 24 hours (ie, within the first day).
- Within the first 3 days.
- Within the first week.
- Within the first 2 weeks.
- Within the first month.
- Within the first 6 weeks.
- Within the first 8 weeks.
- My kitty unfortunately never saw symptom resolution.
- I can't recall.
- Other: _______________

59. Can you clarify how your pet's neurological condition changed throughout treatment, if at all, with approximate weeks different neurological-involved symptoms took to resolve?

60. Have any of your cat's ocular or neurological symptoms persisted in any form, and you or your vet attribute them to permanent or longer-term damage?

Check all that apply.

- [ ] My kitty's walking/gait.
- [ ] My kitty's running.
- [ ] My kitty's jumping.
- [ ] Kitty is unstable at times (for example, only when performing certain motions or startled), but fine others.
- [ ] Kitty has substantial balance issues (for example, hydrocephalus).
- [ ] Kitty dragging of his/her legs and hind leg weakness.
- [ ] Seizures.
- [ ] Tremors or shaking.
- [ ] Twitching.
- [ ] Eye cloudiness/haziness.
- [ ] Uveitis
- [ ] Iritis
- [ ] Pupil unevenness.
- [ ] Blindness.
- [ ] Erratic pupil size.
- [ ] Eyes twitching/darting back and forth (nystagmus).
- [ ] Head tilt.
- [ ] I cannot recall.

Other: [ ] ______________________

61. Did your vet run any tests to determine your pet's neurologic or ocular health? Select all that apply. If you have the test results and are comfortable with sharing, please send them to FIPwarriorsStudy@gmail.com along with bloodwork, as per the question on "report upload" later on.

Check all that apply.

- [ ] FIP or FeCV (coronavirus) test on spinal fluid (ie, a spinal tap).
- [ ] FIP or FeCV (coronavirus) test on fluid from eye (ie, biopsy of aqueous humor).
- [ ] cytology on spinal fluid.
- [ ] cytology on fluid from eye.
- [ ] A physical exam and determined my pet's had a neurological issue.
- [ ] A physical exam and determined my pet's had an eye/ocular issue.
- [ ] Fundic exam (exam of retina for cat).
- [ ] Head magnetic resonance image (head MRI).
- [ ] I cannot recall.

Other: [ ] ______________________

62. Is there anything additional or out of the ordinary you want to share about your cat's symptom neurological or ocular symptom resolution timeline?

____

____

____

____

____

Skip to question 63

Cat Current Condition

63. How is your cat doing? *

Mark only one oval.

- ( ) My cat is alive. *Skip to question 64*
- ( ) My cat unfortunately passed during or after treatment. *Skip to question 118*

Cats still alive

64. Where in treatment is your cat?

Mark only one oval.

- ◯ My cat is being actively treated for a relapse. *Skip to question 68*
- ◯ My cat is in the observation period.
- ◯ My cat is cured. *Skip to question 65*

Observation or Cured cat questions

65. Does your cat have any residual effects that you or your vet attribute to the GS treatment, and not from FIP itself? Please describe. For example, some cats experience scars at the sites of injections.

66. Does your cat still display any residual signs of FIP, that you or your vet attribute to damage from FIP? Please describe. For example, some neurological FIP cats display poor balance, even after being cured of FIP.

Did you cat ever suffer a relapse, or need any additional courses of treatment or extended treatment for relapses?

A relapse may include but is not limited to:
1. A cat that completes treatment and is under observation, but begins to display symptoms again.
2. A cat that has symptoms under control (ie, is showing no or recovering symptoms) and symptoms dramatically worsen.
3. A cat that develops a new form of FIP during treatment (for example, a cat receiving treatment for dry or wet FIP, which develops neurological or ocular symptoms).

67. Please share.

Mark only one oval.

- ◯ Yes *Skip to question 68*
- ◯ No *Skip to question 123*

Skip to question 123

Generic Relapse Questions

A relapse may include but is not limited to:
1. A cat that completes treatment and is under observation, but begins to display symptoms again.
2. A cat that has symptoms under control (ie, is showing no or recovering symptoms), but symptoms dramatically worsen.
3. A cat that develops a new form of FIP during treatment (for example, a cat receiving treatment for dry or wet FIP, which develops neurological or ocular symptoms).

68. Are there any other cats other than your FIP cat in your household, or is your cat outdoors? If so, please explain, particularly focusing on the number of other cats your FIP cat may have had contact with (if known). Finally, did your cat share a litter box or litter area with any of these cats at any point?

____
____
____
____
____

69.

Mark only one oval.

- Option 1

70. How many relapses did your cat face?

Mark only one oval.

- A single relapse. *Skip to question 71*
- Two relapses. *Skip to question 79*
- Three relapses or more. *Skip to question 94*

Single Relapse Extended Weeks

71. When did you notice the relapse?

Mark only one oval.

- During the initial treatment period (typically, 12-weeks).
- During the observation period (the first 84 days post-therapy).
- When my cat had been labelled as cured (after the 84 day observation period).

How many days/weeks were you into the period during which you noticed the relapse?

For example, if you noticed a relapse 12 days after you you finished the first round of treatment, please respond "12 days".

If you noticed the relapse 60 days into the initial treatment period, please respond "60 days".

if you noticed the relapse when your cat was labelled as cured, please respond how many days since the previous administration of GS.

72. Please share.

How many additional weeks was the first relapse treated for?

If the relapse occurred during initial treatment period, this number should reflect the number of weeks you added ON to the number of weeks you initially planned to treat the first relapse. For example, if you initially planned to treat 12 weeks, but had a relapse during the 8th week and ended up going to 16 weeks total, this number would be "4" (16 - 12). If you made no changes to your initial plan of 12 weeks, this number would be "0".

If the relapse occurred during an observation period, this should reflect the number of weeks you treated, or plan to treat, this relapse for.

if you can't recall, please write "I do not recall."

73. Please share.

74. What led to you identifying a relapse?

Check all that apply.

- [ ] Bloodwork issues (ie, globulins increased, A/G dropped, etc).
- [ ] Development or worsening of Behavioral problems (appetite, lethargy, weakness, etc.)
- [ ] Development or worsening of neurological or ocular issues.
- [ ] New or recurrent symptoms.

Other: [ ] ______________________

75. Please describe.

76. What additional actions were taken, if any, to address the relapse? For example, "dosage was increased to 10 mg/kg, and we added proboost therapy for the relapse".

77. Do you remember how long it took to notice the first signs of improvement for the relapse?

78. Are you still in the course of treating the first relapse?

Mark only one oval.

- Yes
- No, we are in the observation period.
- Our kitty passed during the most recent treatment for a relapse.

Skip to question 123

Double Relapse Extended Weeks

79. When did you notice the first relapse?

Mark only one oval.

- ( ) During the initial treatment period (typically, 12-weeks).
- ( ) During the observation period (the first 84 days post-therapy).
- ( ) When my cat had been labelled as cured (after the 84 day observation period).

How many days/weeks were you into the period noted in the previous question were you when you noticed the signs of the first relapse?

For example, if you noticed a relapse 12 days after you you finished the first round of treatment, please respond "12 days".

If you noticed the relapse 60 days into the initial treatment period, please respond "60 days".

if you noticed the relapse when your cat was labelled as cured, please respond how many days since the previous administration of GS.

80. Please share.

How many additional weeks was the first relapse treated for?

If the relapse occurred during initial treatment period, this number should reflect the number of weeks you added ON to the number of weeks you initially planned to treat the first relapse. For example, if you initially planned to treat 12 weeks, but had a relapse during the 8th week and ended up going to 16 weeks total, this number would be "4" (16 - 12). If you made no changes to your initial plan of 12 weeks, this number would be "0".

If the relapse occurred during an observation period, this should reflect the number of weeks you treated, or plan to treat, this relapse for.

81. Please share.

82. Do you remember how long it took to notice the first signs of improvement for the relapse?

83. What factors led to you identifying the first relapse?

Check all that apply.

- [ ] Bloodwork issues (ie, globulins increased, A/G dropped, etc).
- [ ] Development or worsening of Behavioral problems (appetite, lethargy, weakness, etc.)
- [ ] Development or worsening of neurological or ocular issues.
- [ ] New or recurrent symptoms.

Other: [ ] ____________________

84. Please describe.

85. What additional actions were taken, if any, to address the first relapse? For example, "dosage was increased to 8 mg/kg" or "we added proboost therapy".

86. When did you notice the second relapse?

Mark only one oval.

- ◯ During the observation period (the first 84 days post-therapy).
- ◯ During treatment for the first relapse.
- ◯ When my cat had been labelled as cured (after the 84 day observation period).

How many days/weeks were you into the period noted in the previous question were you when you noticed the signs of the second relapse?

For example, if you noticed a relapse 12 days after you you finished the first round of treatment, please respond "12 days".

If you noticed the relapse 60 days into the treatment of the first relapse, please respond "60 days".

if you noticed the relapse when your cat was labelled as cured, please respond how many days since the previous administration of GS.

87. Please share.

How many additional weeks was the second relapse treated for, or do you plan to treat for?

If the relapse occurred during treatment for a prior relapse, this number should reflect the number of weeks you added ON to the number of weeks you initially planned to treat the prior relapse. For example, if you were treating a relapse and initially planned to do 6 weeks, but had a second relapse during the second week and ended up going to 8 weeks total, this number would be "2" (8 - 6). If you made no changes to your initial plan of 6 weeks for the first relapse, this number would be "0".

If the relapse occurred during an observation period, this should reflect the number of weeks you treated, or plan to treat, this relapse for.

if you can't recall, please write "I do not recall."

88. Please share.

89. What factors led to you identifying the second relapse?

Check all that apply.

- [ ] Bloodwork issues (ie, globulins increased, A/G dropped, etc).
- [ ] Development or worsening of behavioral problems (appetite, lethargy, weakness, etc.)
- [ ] Development or worsening of neurological or ocular issues.
- [ ] New or recurrent symptoms.

Other: [ ] ____________________

90. Please describe.

91. What additional actions were taken, if any, to address the second relapse? For example, "dosage was increased to 8 mg/kg" or "we added proboost therapy".

92. Do you remember how long it took to notice the first signs of improvement for the second relapse?

93. Are you still in the course of treating the second relapse?

Mark only one oval.

- Yes
- No, we are in the observation period.
- Our kitty passed during the most recent treatment for a relapse.

Skip to question 123

Three+ Relapse Extended Weeks

94. When did you notice the first relapse?

Mark only one oval.

- ◯ During the initial treatment period (typically, 12-weeks).
- ◯ During the observation period (the first 84 days post-therapy).
- ◯ When my cat had been labelled as cured (after the 84 day observation period).

How many days/weeks were you into the period noted in the previous question were you when you noticed the signs of the first relapse?

For example, if you noticed a relapse 12 days after you you finished the first round of treatment, please respond "12 days".

If you noticed the relapse 60 days into the treatment of the first relapse, please respond "60 days".

if you noticed the relapse when your cat was labelled as cured, please respond how many days since the previous administration of GS.

95. Please share.

How many additional weeks was the first relapse treated for?

If the relapse occurred during initial treatment period, this number should reflect the number of weeks you added ON to the number of weeks you initially planned to treat the first relapse. For example, if you initially planned to treat 12 weeks, but had a relapse during the 8th week and ended up going to 16 weeks total, this number would be "4" (16 - 12). If you made no changes to your initial plan of 12 weeks, this number would be "0".

If the relapse occurred during an observation period, this should reflect the number of weeks you treated, or plan to treat, this relapse for.

if you can't recall, please write "I do not recall."

96. Please share.

97. What factors led to you identifying the relapse?

Check all that apply.

- [ ] Bloodwork issues (ie, globulins increased, A/G dropped, etc).
- [ ] Development or worsening of Behavioral problems (appetite, lethargy, weakness, etc.)
- [ ] Development or worsening of neurological or ocular issues.
- [ ] New or recurrent symptoms.

Other: [ ] ____________________

98. Please describe.

99. What additional actions were taken, if any, to address the first relapse? For example, "dosage was increased to 8 mg/kg" or "we added proboost therapy".

100. Do you remember how long it took to notice the first signs of improvement for the first relapse?

101. When did you notice the second relapse?

Mark only one oval.

- During the observation period (the first 84 days post-therapy).
- During treatment for the first relapse.
- When my cat had been labelled as cured (after the 84 day observation period).

How many days/weeks were you into the period noted in the previous question were you when you noticed the signs of the first relapse?

For example, if you noticed a relapse 12 days after you you finished the first round of treatment, please respond "12 days".

If you noticed the relapse 60 days into the treatment of the first relapse, please respond "60 days".

if you noticed the relapse when your cat was labelled as cured, please respond how many days since the previous administration of GS.

102. Please share.

How many additional weeks was the second relapse treated for, or do you plan to treat for?

If the relapse occurred during treatment for a prior relapse, this number should reflect the number of weeks you added ON to the number of weeks you initially planned to treat the prior relapse. For example, if you were treating a relapse and initially planned to do 6 weeks, but had a second relapse during the second week and ended up going to 8 weeks total, this number would be "2" (8 - 6). If you made no changes to your initial plan of 6 weeks for the first relapse, this number would be "0".

If the relapse occurred during an observation period, this should reflect the number of weeks you treated, or plan to treat, this relapse for.

if you can't recall, please write "I do not recall."

103. Please share.

____

____

____

____

____

104. What factors led to you identifying the relapse?

Check all that apply.

- [ ] Bloodwork issues (ie, globulins increased, A/G dropped, etc).
- [ ] Development or worsening of Behavioral problems (appetite, lethargy, weakness, etc.)
- [ ] Development or worsening of neurological or ocular issues.
- [ ] New or recurrent symptoms.

Other: [ ] ____________________

105. Please describe.

____

____

____

____

____

106. Do you remember how long it took to notice the first signs of improvement for the second relapse?

107. What additional actions were taken, if any, to address the second relapse? For example, "dosage was increased to 8 mg/kg" or "we added proboost therapy".

108. When did you notice the third relapse?

Mark only one oval.

- During the observation period (the first 84 days post-therapy).
- During treatment for the second relapse.
- When my cat had been labelled as cured (after the 84 day observation period).

How many days/weeks were you into the period noted in the previous question were you when you noticed the signs of the third relapse?

For example, if you noticed a relapse 12 days after you you finished the first round of treatment, please respond "12 days".

If you noticed the relapse 60 days into the treatment of the first relapse, please respond "60 days".

if you noticed the relapse when your cat was labelled as cured, please respond how many days since the previous administration of GS.

109. Please share.

____
____
____
____
____

How many additional weeks was the third or more relapse(s) treated for, or do you plan to treat for?

If the relapse occurred during treatment for a prior relapse, this number should reflect the number of weeks you added ON to the number of weeks you initially planned to treat the prior relapse. For example, if you were treating a second relapse and initially planned to do 6 weeks, but had a third relapse during the second week and ended up going to 8 weeks total, this number would be "2" (8 - 6). If you made no changes to your initial plan of 6 weeks for the second relapse, this number would be "0".

If the relapse occurred during an observation period, this should reflect the number of weeks you treated, or plan to treat, this relapse for.

if you can't recall, please write "I do not recall."

110. Please share.

____
____
____
____
____

111. What factors led to you identifying the most third (and later) relapses?

Check all that apply.

- [ ] Bloodwork issues (ie, globulins increased, A/G dropped, etc).
- [ ] Development or worsening of Behavioral problems (appetite, lethargy, weakness, etc.)
- [ ] Development or worsening of neurological or ocular issues.
- [ ] New or recurrent symptoms.

Other: [ ] ________________________

112. Please describe.

113. What additional actions were taken, if any, to address the third (or additional) relapses? For example, "dosage was increased to 8 mg/kg" or "we added proboost therapy".

114. Do you remember how long it took to notice the first signs of improvement for the additional relapse(s)?

115. How many additional weeks were any additional relapse courses treated for? Please describe.

116. Are you still in the course of treating the most recent relapse?

Mark only one oval.

- Yes
- No, we are in the observation period.
- Our kitty passed during the most recent treatment for a relapse.

117. How many relapses, in total, did your cat experience?

Skip to question 123

Cats that did not survive

118. If you did start GS therapy with your cat, how many days after your cat became ill (Showed the first clinical signs) did you begin therapy?

119. When during treatment did your cat pass?

Mark only one oval.

- ◯ During the first round of treatment
- ◯ During treatment for a relapse
- ◯ During the observation period.
- ◯ After being denoted as cured.
- ◯ My cat did not begin therapy
- ◯ Other: ____________________

120. If you did start GS therapy with your cat, how many days of therapy were you able to give before your cats passing? If you finished the 84 days (12 weeks) and then your cat had a relapse, please indicate that as well.

121. Did you or your vet have any thoughts as to how or why your cat might have passed? For example, what was the cause of death, and the symptoms the precipitated your cat's unfortunate passing?

Did you cat ever suffer a relapse, or need any additional courses of treatment or extended treatment for relapses?

A relapse may include but is not limited to:
1. A cat that completes treatment and is under observation, but begins to display symptoms again.
2. A cat that has symptoms under control (ie, is showing no or recovering symptoms) and symptoms dramatically worsen.
3. A cat that develops a new form of FIP during treatment (for example, a cat receiving treatment for dry or wet FIP, which develops neurological or ocular symptoms).

122. Please share.

Mark only one oval.

- Yes. *Skip to question 68*
- No. *Skip to question 123*

Closing Thoughts

123. Did you experience difficulty at home administering the GS therapy?

Mark only one oval.

- Yes
- No

124. If you experienced difficulty administering the GS therapy, did your veterinarian or a member of their staff assist you in giving the medication?

Mark only one oval.

- Yes
- No

125. Did your cat experience any of these signs that was attributed the the GS therapy? Check all that apply.

Check all that apply.

- [ ] Vocalization with injection
- [ ] Pain at injection site
- [ ] My cat would not hold still while administering medication.
- [ ] Salivation after oral administration of GS medication
- [ ] Vomiting
- [ ] Diarrhea
- [ ] Increased appetite
- [ ] Decreased appetite
- [ ] Increased activity level
- [ ] Decreased activity level
- [ ] Swelling at injection site
- [ ] Bleeding at injection site
- [ ] Open wound at an injection site
- [ ] Infection at injection site

Other: [ ] ____________________

126. What other supportive therapies were utilized by you or your veterinarian to treat your cat (in addition to the GS therapy) during the treatment period?

Check all that apply.

- [ ] Prednisolone/Prednisone/oral steroids
- [ ] Depomederol/injectable steroids
- [ ] Mirtazapine/Mirtaz
- [ ] Vitamin B12 injections
- [ ] Antibiotics
- [ ] Cerenia (for nausea)
- [ ] Subcutaneous fluids
- [ ] IV fluids
- [ ] T-cyte/Proboost/Thymic Protein A supplementation
- [ ] Blood Transfusions
- [ ] Any form of light therapy (laser skin therapy, etc.)

Other: [ ] ____________________

127. If your cat was on prednisolone or other steroids (injectable or oral) at all during therapy, how long was it until you discontinued steroid administration?

Mark only one oval.

- This question does not apply to my cat.
- We discontinued steroids prior to starting GS therapy.
- The first day.
- Within the first 3 days.
- Within the first week.
- Within the first 2 weeks.
- Within the first month.
- Within the first 2 months.
- We discontinued therapy in the beginning of treatment, but added it back later.
- We never discontinued steroid therapy.
- Other: ______________________

128. What else would you like to tell us, that we have not asked?

This content is neither created nor endorsed by Google.

Google Forms
